# Quercetin reduces tendon adhesion in rat through suppression of oxidative stress

**DOI:** 10.1186/s12891-020-03618-2

**Published:** 2020-09-11

**Authors:** Yuan Liang, Keteng Xu, Pei Zhang, Jiale Zhang, Pengtao Chen, Jinshan He, Yongchao Fang, Yuelai Zhou, Jingcheng Wang, Jianzhong Bai

**Affiliations:** 1 Department of Orthopedics, Clinical Medical College of Yangzhou University, Subei People’s Hospital, Nantong West Road 98, Yangzhou, 225001 China; 2 Department of Orthopedics, The Second Xiangya Hospital of Central South University, Changsha, 410011 Hunan China; 3 Department of Orthopedics, The Second Affiliated Hospital of Bengbu Medical College, Bengbu, 233000 China

**Keywords:** Quercetin, Tendon adhesions, Oxidative stress

## Abstract

**Background:**

Tendon adhesion is one of the most common clinical problems, which poses a considerable challenge to orthopedics doctors. Quercetin (QUE) as a popular drug at present, it has various biological functions, including anti-inflammatory, anti-ischemic, anti-peroxidation, and antioxidant. The purpose of this study was to investigate the effect of quercetin on tendon adhesion and whether quercetin can inhibit oxidative stress.

**Method:**

Thirty-six rats were randomly divided into three groups, including control group, low QUE (50 mg/kg/day) group, and high QUE (100 mg/kg/day) group. After 1 week, the levels of SOD, MDA and GPx were measured. The degree of tendon adhesion was assessed by macroscopic evaluation and histological evaluation. After 4 weeks. Besides, the pharmacological toxicity of quercetin to main organs were evaluated by histological analysis.

**Results:**

The extent of superoxide dismutase (SOD) and glutathione peroxidase (GPx) of tendon tissue in high QUE group was significantly higher than those of low QUE group and control group. And the extent of malondialdehyde (MDA) of tendon tissue in high QUE group was significantly lower than that of low QUE group and control group. By macroscopic evaluation and histological analysis, the extent of tendon adhesion in high QUE group was lower than low QUE group and control group. However, there were no significant changes of the major organs through histological analysis.

**Conclusions:**

Quercetin may be a good and safe strategy in preventing tendon adhesion. But further clinical research is needed before its recommendation in the prevention and treatment of tendon adhesion.

## Background

Tendon adhesion is a critical clinical issue characterized by the limitation of tendon gliding, which poses a considerable challenge to orthopedic doctors [1, 2]. Two pathways are considered to participate in tendon repair. The intrinsic repair pathway involves the internal repair function of tenocytes, the extrinsic repair process involves the invasion of cells from the outside of tendon and finally promotes the formation of adhesion [3, 4]. As the pathophysiological mechanism of tendon adhesion is not clear, the prevention and treatment of tendon adhesion is still facing challenges. Many methods have been used to prevent tendon adhesion, such as the improvement of surgical technology, the use of barriers, systemic or local application of drugs and chemicals. Although these methods can reduce adhesions to some extent, the residual or recurrent adhesions still need to be resolved [5–8].

Reactive oxygen species (ROS) as essential mediators of fibrogenesis, it is possible that ROS also play a role in the formation of tendon adhesion [9]. In addition, some studies have demonstrated that antioxidation strategies could reduce the extent of tendon adhesion by reducing ROS [10]. QUE is an abundant nature flavonoid which could be found in many fruits, vegetables, leaves and grains. It has been reported that QUE has the biological functions of anti-inflammation, anti-ischemia, anti-oxidation and anti-tumor [11–14]. Also, QUE is a strong antioxidant and radical scavenger [15–17]. Therefore, we considered it may be a functional drug to treat tendon adhesion, thus, we conducted rat models to figure out the effect of QUE on tendon adhesion.

## Methods

### Ethical consent

The animal experiments were carried out in accordance with the guide of the Clinical Medical College of Yangzhou University (Yangzhou, China).

### Chemicals

Quercetin was purchased from Dalian Meilun Biological Technology Co., Ltd. (Dalian, China). SOD, GPx and MDA detection kits were purchased from Nanjing Jiancheng Biotechnology Limited (Nanjing, China).

### Animal preparation

Thirty-six Male Wistar rats weighting 200 to 220 g were purchased from Yangzhou University (Yangzhou, China). The rats were enrolled equally into three groups, including control group, Low QUE (50 mg/kg/day) group, and High QUE (100 mg/kg/day) group. The doses were selected according to several published studies on the use of QUE in rats [18–21]. All rats were transferred to the laboratory 1 week before the study.

The animal model of tendon adhesion was established based on previous study [22]. Rats were anesthetized by intraperitoneal injection of 2% Pentobarbital Sodium (25 mg / kg) and fixed on the operating table. The right hind limb of each rat was shaved and scrubbed with povidone iodine. A longitudinal incision about 2 cm long were made on the inside of the plantar skin. Next, the subcutaneous tissue was dissected and the tendon was cut through by using 11# scalpels. The tendon was sutured with 5–0 nylon by modified Kessler suture technique. The skin incision was closed with 3–0 nylon. All rats were injected with penicillin 1,600,000 U / kg to prevent infection. The rats were kept in separate cages and were free to get food and water.

### Biochemical study

Six rats of each group were randomly selected and sacrificed with overdose of pentobarbital at day 7. Tendon tissues were stored at − 80 °C until homogenized to analysis. The levels of malondialdehyde (MDA), superoxide dismutase (SOD) and glutathione peroxidase (GPx) activities were measured using different commercial assay kits.

### Macroscopic evaluation

The other six rats of each group were killed with excessive pentobarbital 4 weeks after operation. The limbs were cut through the original incision. Two independent pathologists made blind observation on the experiment according to the previously reported methods: Level 1: no adhesion; Level 2: blunt peeling can separate adhesion; Level 3: sharp peeling is required, and no more than 50% of the adherent tissues are separated; level 4: sharp peeling can separate 51–97.5% of the adherent tissues; level 5: sharp peeling can separate more than 97.5% of the adherent tissues [23, 24]. When disagreement existed, it was resolved by consulting another pathologist.

### Histological analysis

After macroscopic evaluation, tendon tissues were used for histological analysis. The samples were fixed in 4% paraform 24 h, and then embedded in paraffin. Four successive transversal sections of four-micrometer were obtained. Two odd sections of each group were stained with hematoxylin and eosin (H & E) and the adhesions were evaluated under the light microscope with the magnification × 200. Two even sections of each group were stained with Masson’s trichrome and the collagen density of adhesion tissue was evaluated at 200× magnification. All sections for an individual case were counted blinded to the group assignment.

### Statistical analysis

The data were analyzed using SPSS 22.0. The significance of differences was calculated by using one-way analysis of variance (ANOVA) followed by Tukey for multiple comparisons. *P* < 0.05 was considered as statistically significant.

## Results

The surgery was well tolerated by all rats. There was no wound infection and mortality during the experiment.

### Effect of treatment of QUE on antioxidant enzyme activities

**Fig. 1:**
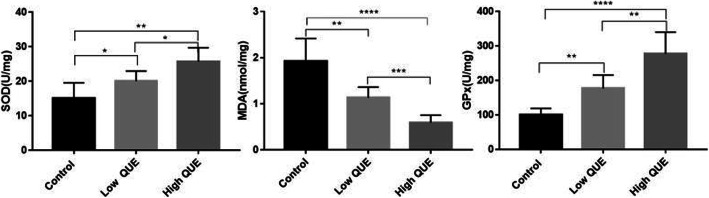
The effect of QUE on oxidative stress-related products of tendon simples at day 7 after surgery. Six rats of each group were randomly selected. Quercetin decreased levels of MDA and elevated activity of SOD and GSH-Px. *P < 0.05; **P < 0.01; ***P < 0.001. Low QUE; 50 mg/kg/day; High QUE, 100 mg/kg/day

As shown in Fig. 1, QUE significantly increased the activities of SOD (Control: 15.02 ± 1.844, Low QUE: 20 ± 1.202, High QUE: 25.68 ± 1.643) and GPx (Control: 99.96 ± 7.744, Low QUE: 176.9 ± 15.81, High QUE: 277 ± 25.61). The MDA levels were significantly lower in the QUE groups (Control: 1.92 ± 0.203, Low QUE: 1.13 ± 0.09448, High QUE: 0.5917 ± 0.06524). Besides, our results showed the antioxidant capacity of QUE was dose-dependent.

### Macroscopic evaluation of tendon adhesion

Macroscopic observation showed that there was weak or moderate adhesion in quercetin treated group. However, in control group, thick fibrous adhesions were observed. The degree of tendon adhesions was evaluated according to the above method **(**Table 1**).**

**Table 1:** Gross adhesion scores in different groups

Group	Grade				
	1	2	3	4	5
control			2	3	1
Quercetin (50 mg/kg)		3	2	1	
Quercetin (100 mg/kg)	1	4	1		

### Effect of QUE on tendon adhesion in histological analysis

**Fig. 2:**
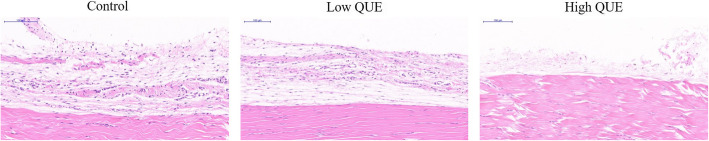
The Effect of QUE on tendon adhesion in histological analysis. The loose scar tissue was found around the of tendon surgical areas treated with 100 mg/kg quercetin group. Mild scar tissues were observed in 50 mg/kg quercetin-treated group. Dense scar tissue was found in the control group. The sections were stained with HE (200×). Low QUE, 50 mg/kg/day; High QUE, 100 mg/kg/day

In control group, dense scar adhesions were found around the surgical areas. In 50 mg/kg quercetin-treated groups, mild scar tissues were observed around the surgical compared with those of control group. However, no or loose fibrous adhesion tissue were observed in 100 mg/kg quercetin-treated group (Fig. 2).

### Effect of QUE on collagen density of tendon

**Fig. 3:**
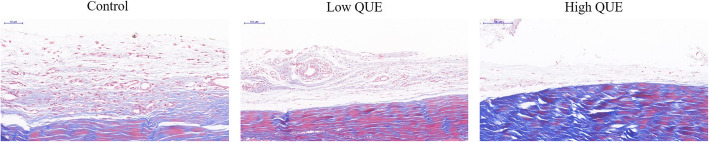
The effect of QUE on tendon collagen density. The collagen tissues show blue in the section with Masson’s trichrome staining under the light microscope (200×). QUE could reduce collagen synthesis and fibrosis. The density of collagen tissue in 100 mg/kg quercetin-treated group seemed to be lower than those in 50 mg/kg quercetin-treated group and control group. Low QUE, 50 mg/kg/day; High QUE, 100 mg/kg/day

In Masson’s trichrome staining, collagen density of tendon adhesion tissue in quercetin-treated groups was coincidence with HE staining. The collagen density of tendon tissue in control group was dense. However, the collagen density was weak in 100 mg/kg quercetin-treated groups, which revealed decrease compared with those in 50 mg/kg quercetin-treated groups. Moreover, the collagen density was moderate in 50 mg/kg quercetin-treated groups, which was also revealed decrease compared with that in control group (Fig. 3).

### Pilot toxicity study

**Fig. 4:**
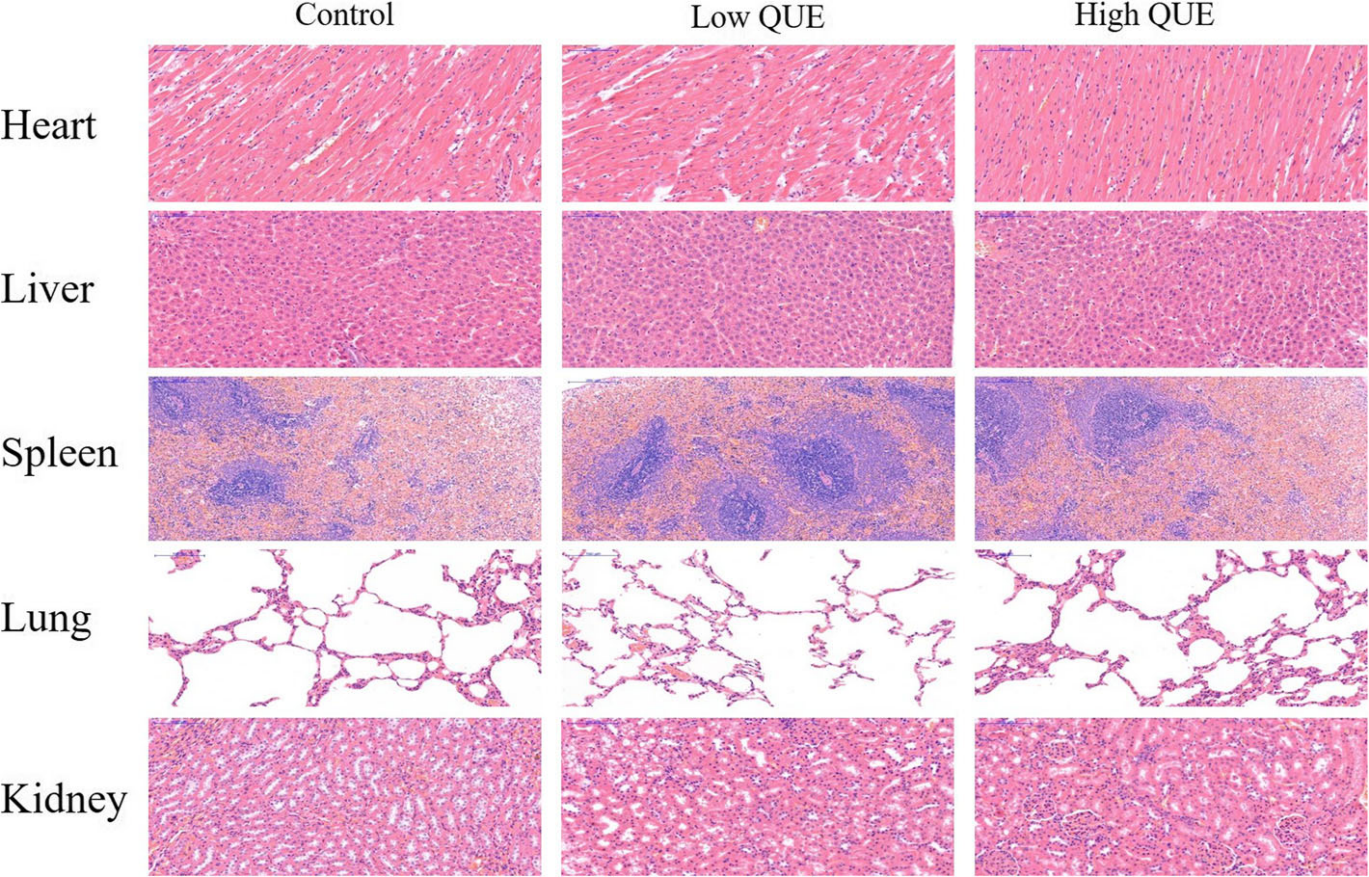
The potential toxicity of QUE in vivo. HE-stained tissue sections of major organs (including heart, liver, spleen, lung, and kidney) from different groups. Low QUE, 50 mg/kg/day; High QUE, 100 mg/kg/day

To evaluate the potential toxicity of QUE in vivo. The toxicity to major organs (Heart, liver, spleen, lung and kidney) was investigated by HE staining (Fig. 4). The histological analysis indicated that QUE did not show any distinct changes of the major organs at the end of this study. All these results demonstrated that our developed therapeutic strategy was effective and safe.

## Discussion

Our study demonstrated that QUE could increase SOD and GPX, and reduce MDA in a dose-dependent manner. Besides, the degree of tendon adhesion in high QUE group was lower than low QUE group and control group. Furthermore, the histological analysis of main organs showed that quercetin has no obvious toxicity.

Tendon adhesion mostly occurs after tendon surgery and joint immobilization. Several mechanisms have been implicated in the modulation of adhesion formation, including the regulation of the TGF-β signaling pathway, oxidative stress, and the inflammatory response [3, 25].

ROS is mainly produced by mitochondria, which plays a dual role in the process of cell physiology. The low level of ROS can regulate specific signal pathways, and too many ROS participate in the pathogenesis of many fibrotic diseases such as tendon adhesion [26–28]. It can be attenuated by various antioxidant enzymes such as SOD and GPx, which could indirectly reflect the oxidative state [29, 30]. Malondialdehyde (MDA, a marker of lipid peroxidation) is usually used as a biomarker of oxidative damage [31]. Increased MDA production and oxidative stress are able to accelerate the accumulation of ECM components and the proliferation of interstitial fibroblasts. In addition, more and more evidences have strongly implicated MDA production and oxidative stress in the pathological of fibrosis [32]. Quercetin is one of the most prevalent plant flavonoids contained in many fruits and vegetables. Within the flavonoid family, quercetin is the most potent scavenger of ROS [33, 34]. Previous studies reported that quercetin could ameliorate pulmonary fibrosis, renal fibrosis, liver fibrosis [35–37].

In current study, we collected tendon samples to measure MDA, SOD and GPx after 1 week of surgery. The results showed that QUE significantly increased the activities of SOD (Control: 15.02 ± 1.844, Low QUE: 20 ± 1.202, High QUE: 25.68 ± 1.643) and GPx (Control: 99.96 ± 7.744, Low QUE: 176.9 ± 15.81, High QUE: 277 ± 25.61). While, the MDA levels were significantly decreased in the QUE groups (Control: 1.92 ± 0.203, Low QUE: 1.13 ± 0.09448, High QUE: 0.5917 ± 0.06524), and the effect was dose-dependent. To further the research, we made macroscopic evaluation and histological analysis of the tendon 4 weeks after the operation. Compared with the control group, the adhesion of tendon in quercetin group was relieved obviously, and the effect of reducing adhesion was better with the increase of quercetin dose, which is consistence with the results of measurement of SOD, GPx, fMDA.

Interestingly, we did not see any significant adverse effects in terms of signs of infection and wound healing. Considering the possibility of the potential toxicity for the major organs, we also investigated the major organs by HE staining. Fortunately, histological analysis also demonstrated that quercetin didn’t show any pilot toxicity.

## Conclusion

Quercetin may be a good and safe strategy in preventing tendon adhesion. But further clinical research is needed before its recommendation in the prevention and treatment of tendon adhesion.

## Data Availability

The datasets used and/or analyzed during the current study are available from the corresponding author upon reasonable request.

## Abbreviations

QUE: Quercetin
SOD: Superoxide dismutase
GPx: Glutathione peroxidase
MDA: Malondialdehyde
ROS: Reactive oxygen species
